# Could Neutrophil-to-Lymphocyte Ratio (NLR) Serve as a Potential Marker for Delirium Prediction in Patients with Acute Ischemic Stroke? A Prospective Observational Study

**DOI:** 10.3390/jcm8071075

**Published:** 2019-07-22

**Authors:** Katarzyna Kotfis, Marta Bott-Olejnik, Aleksandra Szylińska, Iwona Rotter

**Affiliations:** 1 Department of Anesthesiology, Intensive Therapy and Acute Intoxications, Pomeranian Medical University, 71-204 Szczecin, Poland; 2 Neurology Department of a Regional Specialist Hospital in Gryfice, 72-300 Gryfice, Poland; 3 Department of Medical Rehabilitation and Clinical Physiotherapy, Pomeranian Medical University, 71-204 Szczecin, Poland

**Keywords:** NLR, CRP, NIHSS, leucocytes, lymphocytes, neutrophils, acute brain dysfunction

## Abstract

Delirium is an acute brain disorder that commonly occurs in patients with acute ischemic stroke (AIS). Pathomechanism of delirium is related to the neuroinflammatory process and oxidative stress. Search for readily available diagnostic marker that will aid clinicians in early identification of delirium is ongoing. The aim of this study was to investigate whether neutrophil-to-lymphocyte ratio (NLR) could serve as a potential marker for delirium prediction in patients with AIS and to find an easy diagnostic tool using laboratory and clinical parameters to predict delirium. Prospective observational study (NCT03944694) included patients with AIS admitted to the neurology department of a district general hospital. All patients were screened for delirium using CAM-ICU (Confusion Assessment Method for Intensive Care Unit). Demographic and medical history data and admission lab results, including differential white blood cell analysis, were collected from all patients. We included 1001 patients in the final analysis. The mean age of the sample was 71 years, and 52% of patients were males. The incidence of early-onset delirium was 17.2%. The NLR was elevated in delirious patients (6.39 ± 8.60 vs. 4.61 ± 5.61, *p* < 0.001). The best cut-off value of NLR to predict delirium using the receiver operating characteristics (ROC) was determined at 4.86. Multivariable logistic regression analysis showed that the odds ratio (OR) for developing delirium with NLR > 4.86 (adjusted for age, sex, body mass index (BMI), comorbidities, and baseline neurology) was 1.875 (95% CI 1.314–2.675, *p* = 0.001). As a result of different combinations of markers and clinical parameters based on logistic regression, a formula—DELirium in Acute Ischemic Stroke (DELIAS score)—was obtained with the area under the ROC curve of 0.801 (*p* < 0.001). After regression of the cut-off points of the obtained curve, a significant correlation of the DELIAS score was observed with the occurrence of early-onset delirium (OR = 8.976, *p* < 0.001) and with delirium until the fifth day after AIS (OR = 7.744, *p* < 0.001). In conclusion, NLR can be regarded as a potential marker for prediction of early-onset delirium after AIS. On the basis of combined laboratory and clinical parameters, the DELIAS score was calculated, which gave the highest predictive value for delirium in the analyzed group of patients after ischemic stroke. However, further studies are needed to validate these findings.

## 1. Introduction

Delirium is an acute neuropsychiatric syndrome that often presents as a complication of generalized infection, hypoxia, surgery, effect of various medications, or acute stroke, especially in elderly patients [1,2,3]. It is characterized by rapid onset, significant fluctuation of symptoms throughout the day, disturbance of the wake and sleep cycle, as well as changes in thinking, memory, and behavior [4]. Post-stroke delirium is a well-described medical problem with long-term consequences of increased mortality and morbidity [5]. The incidence of delirium in stroke patients ranges from 12% to 66%, depending on the diagnostic method used [6,7,8]. A recently published prospective delirium study on ischemic brain stroke showed that the delirium frequency was 14.6% in the first week after admission [9]. In the Polish population, delirium associated with acute ischemic stroke (AIS) was diagnosed in approximately 30% of patients [10,11]. 

It is recommended that delirium is actively screened for with dedicated psychometric tools, including CAM-ICU (Confusion Assessment Method for Intensive Care Unit) and ICDSC (Intensive Care Delirium Screening Checklist) [1,12,13]. Mitasova et al. reported that an episode of delirium was detected in 43% of patients with stroke using CAM-ICU monitoring [14]. Delirium in stroke patients may be regarded as more difficult to diagnose and differentiate from new-onset neurological symptoms. However, the validity of CAM-ICU to diagnose delirium in ischemic stroke patients has been confirmed by multiple studies [14,15].

Delirium is a clinical diagnosis, but a search for easily accessible and reliable serum biomarkers of this acute brain dysfunction is ongoing [16]. The pathophysiology of delirium is not entirely understood, but many interrelated disorders of homeostasis are involved, including neuroinflammation and the effect of oxidative stress [17,18]. Considering the inflammatory pathomechanism of delirium, researchers are looking for new diagnostic and prognostic biomarkers in the group of inflammatory factors. However, research in this area is extremely limited, and the results are conflicting. Inflammatory markers investigated in scientific studies have been associated with delirium, but their use in clinical practice is precluded by price and the difficulty of the diagnostic process. Simple laboratory parameters, easily available in every health care facility, performed in all patients at admission are in renaissance at the moment. Differential white blood cell count can be done with the use of any automated hematology analyzer as part of routine blood tests performed at admission. 

Immune system activation seems to be the basis for delirium in acute neurological disorders, such as neuroinfections, trauma, and stroke. The neutrophil-to-lymphocyte ratio (NLR), which is a parameter derived from the differential white blood cell count, is a readily available marker of both inflammation and oxidative stress. It has recently been reported by Egberts at al. to be increased in elderly patients with delirium [19]. 

In many recent studies, the NLR has been used and validated as an inflammatory marker, especially in cardiovascular disorders and malignancies. Celikbilek et al. showed that NLR in peripheral blood can serve as a simple systemic inflammatory response (SIR) marker that possesses a diagnostic value in certain diseases characterized by systemic or local inflammatory response [20]. NLR is an indicator of the overall inflammatory status of the body, such as diabetes mellitus, coronary artery disease (CAD), ulcerative colitis, or inflammatory arthritis [21,22,23,24]. Studies have shown that the NLR index is a good predictor of outcome in neurological and psychiatric conditions, such as Alzheimer’s disease, Parkinson’s disease, ischemic stroke, schizophrenia, and memory disorders, or after endarterectomy of the carotid arteries [25,26,27,28].

The aim of this study was to investigate whether NLR could serve as a potential marker for delirium prediction in patients with AIS and to find an easy diagnostic tool using laboratory and clinical parameters to predict delirium.

## 2. Materials and Methods

### 2.1. Data Collection

A retrospective analysis of prospectively collected data was carried out on 1022 consecutive adult patients (age > 18 years) with acute ischemic stroke that were admitted to the neurology department of a busy district general hospital in Poland between 30 June 2015 and 31 March 2018. Patients with AIS were admitted to the neurology department within 48 hours of symptom development. The data was prospectively collected by a dedicated member of staff and included the following: complete past medical history and clinical and neurological evaluation, stroke severity according to the National Institute of Health Stroke Scale (NIHSS), degree of disability according to the modified Rankin scale (mRS), carotid artery ultrasonography, brain computed tomography (CT), and laboratory results, including full blood count (with differential white blood cell count) and C-reactive protein (CRP). All patients underwent routine blood analysis at admission using a fully automated cell counter in the local hematology laboratory to acquire complete blood count, including absolute neutrophil count, absolute lymphocyte count, and platelet count. The NLR was calculated by dividing the absolute neutrophil count over the absolute lymphocyte count from the same sample. We excluded patients with hematology disorders (5 patients), incomplete laboratory testing (6 patients), or no data regarding follow-up (10). The follow-up data was extracted from medical records and using a telephone contact at 30 days and 1 year after discharge to determine mortality at predefined timepoints. The primary end point was delirium diagnosed within 24 hours from admission in patients with acute ischemic stroke. 

### 2.2. Delirium Monitoring

We used the Polish version of the CAM-ICU assessment tool to screen all patients for delirium at admission and on a daily basis after admission to the hospital [13]. To aid delirium diagnosis, a review of medical and nursing notes for a full evaluation of delirium was performed by one of the investigators. Delirium was diagnosed according to the Diagnostic and Statistical Manual of Mental Disorders 5th edition [4]. Delirium was defined as “early onset” if it was diagnosed within the first 24 hours after admission to the neurology unit due to acute ischemic stroke. 

### 2.3. Ethical Consideration

The study was performed in accordance with the Declaration of Helsinki. We obtained approval of the Bioethical Committee of the Pomeranian Medical University, no. KBE-0012/84/03/19. The requirement for informed consent was waived as all data collected in the study was part of a routine clinical process performed in every AIS patient at admission to the hospital. The data was analyzed anonymously. This study was retrospectively registered at the ClinicalTrials.gov website, identifier number NCT03944694.

### 2.4. Statistical Analysis

The data are presented as mean and standard deviation for continuous variables and number and a percentage for categorical variables. We divided the acute ischemic stroke patients according to the absence or presence of delirium on admission (no delirium group vs. delirium group). Chi-square test or chi-square test with Yates correction was used to compare qualitative data between two groups of patients. The Mann–Whitney *U* test was used to compare continuous variables. The receiver operating characteristic (ROC) curve analysis was performed to determine the best cut-off value for predicting the clinical end points. Logistic regression analysis was performed, with additional correction for potentially interfering variables (age, sex, and body mass index (BMI)). On the basis of this regression, the parameters most closely related to the occurrence of delirium were sought. Based on a multifactorial model, information on the impact of each variable on delirium was obtained. Next, the contribution of each analyzed variable was calculated, and the formula was presented using elements with which the delirium score was calculated. *p*-value ≤ 0.05 was considered significant. All analyses were performed using licensed software Statistica 13 (StatSoft, Inc., Tulsa, OK, USA).

## 3. Results

We included 1001 patients in the final analysis with a complete set of data. The mean age of the sample was 71 years, with 52% of patients being men. The incidence of delirium on admission was 17.2% (172/1001 patients), and we therefore performed a comparative analysis between the subgroups (delirium vs. no delirium). Patients with delirium were older (*p* < 0.001), more often presented with coexisting ischemic heart disease (*p* = 0.003), previous ischemic stroke (*p* = 0.017), impaired glucose tolerance (*p* < 0.001), atrial fibrillation (*p* < 0.001), and peripheral vascular disease (*p* < 0.001), as shown in Table 1.

Neurological findings in the study group are shown in Table 2. Patients with delirium were more severely ill on admission, with higher NIHSS score (18.00 (12.00–21.50) vs. 8.00 (4.00–14.00), *p* < 0.001) and Rankin score (5.00 (4.00–5.00) vs. 3.00 (1.00–4.00), *p* < 0.001). Delirious patients presented with more amaurosis fugax (73.84% vs. 29.19%, *p* < 0.001) and dysphasia (65.70% vs. 52.35%, *p* = 0.001) and more often had lesions >2.5 cm (*p* < 0.001). A majority of delirious patients presented with stroke from middle cerebral artery area (82.35% vs. 62.56%, *p* < 0.001) and 50–70% carotid artery stenosis (38.41% vs. 20.99%, *p* < 0.001).

The outcome data is shown in Table 3. Patients presenting with delirium had longer hospital stay (10.00 (8.00–14.00) days vs. 9.00 (8.00–11.00) days, *p* < 0.001) and higher mortality at day 7 (15.7% vs. 4.95%, *p* < 0.001), at day 30 (38.4% vs. 12.2%, *p* < 0.001), and at one year (23.8% vs. 61.2%, *p* < 0.001). A smaller percentage of AIS patients were discharged home (41.3% vs. 61.9%, *p* < 0.001) or to a rehabilitation center (17.4% vs. 21.1%, *p* < 0.001) if they had delirium on admission. 

Mean levels and standard deviations of laboratory markers adjusted for age and sex are shown in Table 4. In delirious patients, the total leucocyte count was higher (*p* = 0.006), as was the neutrophil count (*p* < 0.001), but the lymphocyte count was lower (*p* = 0.006), giving a higher neutrophil-to-lymphocyte ratio (3.92 (2.44–7.87) vs. 3.14 (2.06–5.09), *p* < 0.001). The CRP level was also higher in delirious patients (7.58 (2.25–30.00) vs. 3.00 (1.10–9.69), *p* < 0.001).

**Figure 1:**
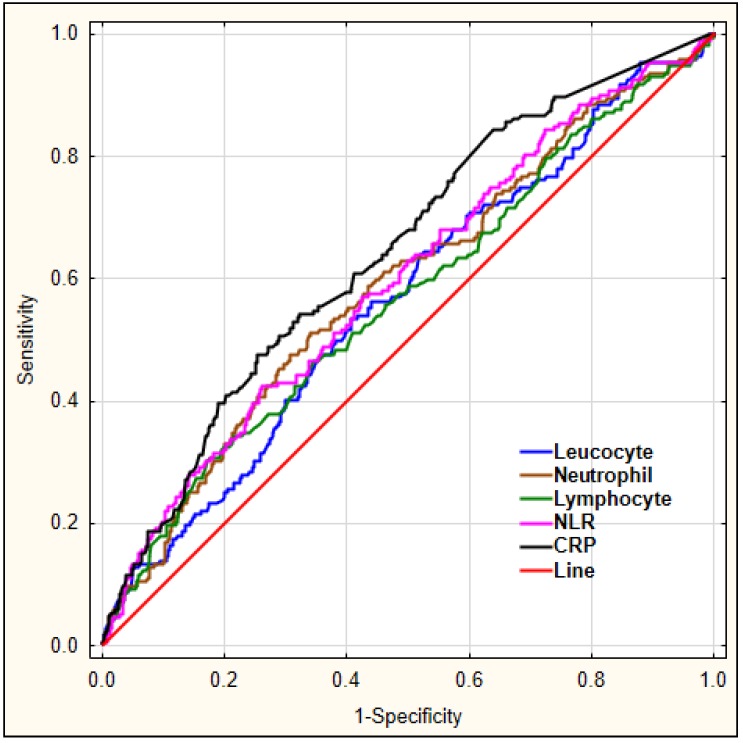
The diagnostic characteristics of leucocyte, neutrophil, lymphocyte count, NLR, and CRP for predicting early-onset delirium in AIS patients. Legend: NLR—neutrophil-to-lymphocyte ratio, CRP—C-reactive protein.

Our aim was to investigate which laboratory markers could serve as diagnostic markers for delirium. For this, we performed a ROC analysis for leucocyte, neutrophil, lymphocyte count, the derived parameter NLR, and CRP to predict early-onset delirium in AIS patients, as shown in Figure 1. 

The details regarding area under the curve (AUC), sensitivity, and specificity for each marker are shown in Table 5.

**Figure 2:**
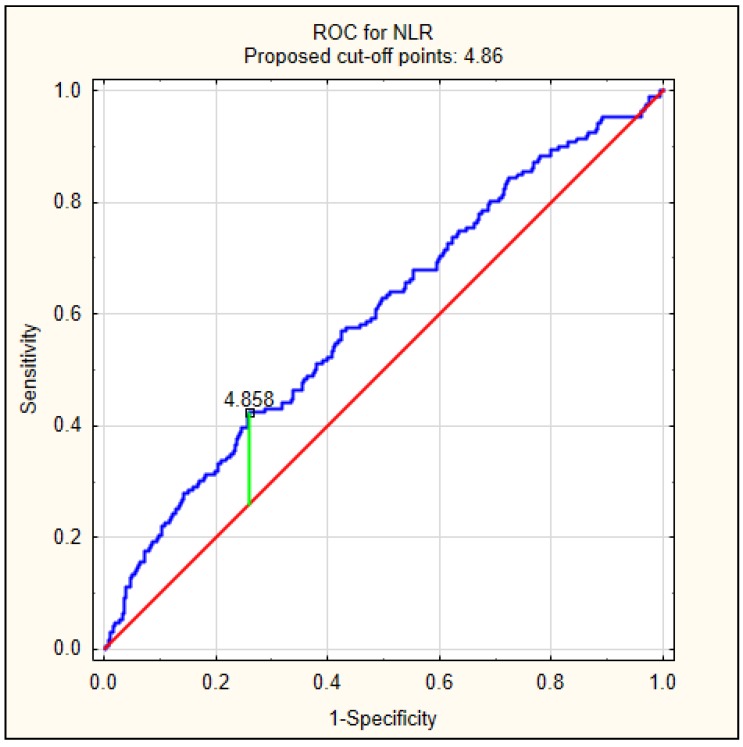
The diagnostic characteristics of NLR for predicting delirium on admission in AIS patients. The best cut-off value of NLR to predict delirium using the ROC was determined at 4.86, with area under the curve of 0.597 (95% CI 0.549–0.644, *p* < 0.001).

Because patients with delirium had significantly higher values of NLR at admission, we decided to perform a ROC analysis to calculate the best cut-off value to predict delirium, as shown in Figure 2. The best cut-off value was 4.86, with an AUC of 0.597 (95% CI 0.549–0.644, *p* < 0.001).

Multivariable logistic regression analysis adjusted according to age, sex, BMI, comorbidities, and baseline neurology showed that leucocyte count (*p* < 0.001) and neutrophil count (*p* = 0.012) as well as mean NRL (*p* = 0.028), NRL (*p* < 0.001), and NLR at the predefined cut-off of >4.86 (*p* < 0.001) exhibited an association with post-stroke delirium after adjustment with baseline characteristics (Table 6). For NLR > 4.86 adjusted for age, sex, BMI, comorbidities, and baseline neurology, the odds ratio (OR) was 1.875 (95% CI 1.314–2.675, *p* = 0.001). Similar finding was noted regarding the CRP, with the cut-off >9.10, for which the OR adjusted for age, sex, BMI, comorbidities, and baseline neurology was even higher with NLR at 2.132 (95% CI 1.482–3.066, *p* < 0.001).

The AUC value for NLR as well as its sensitivity and specificity were moderate, so we decided to find a combination of markers and clinical parameters that would help predict the occurrence of early-onset delirium in AIS. Using clinical and laboratory factors, we determined that an index composed of age, NIHSS score, neurological findings, leucocyte count, NLR, and CRP was better at predicting early-onset delirium after acute ischemic stroke than any of the factors alone. To increase the diagnostic value of the laboratory markers in the study group, clinical and laboratory variables most associated with delirium were determined based on logistic regression. We calculated the score based on logistic regression for delirium in the whole study group as follows: 

DELirium in Acute Ischemic Stroke (DELIAS) score = (1.272 × hemianopia) + (0.098 × aphasia) + (0.026 × age) + (0.054 × NIHSS score on admission) − (0.005 × NLR) + (0.028 × Leukocytes) + (0.001 × CRP).

**Figure 3:**
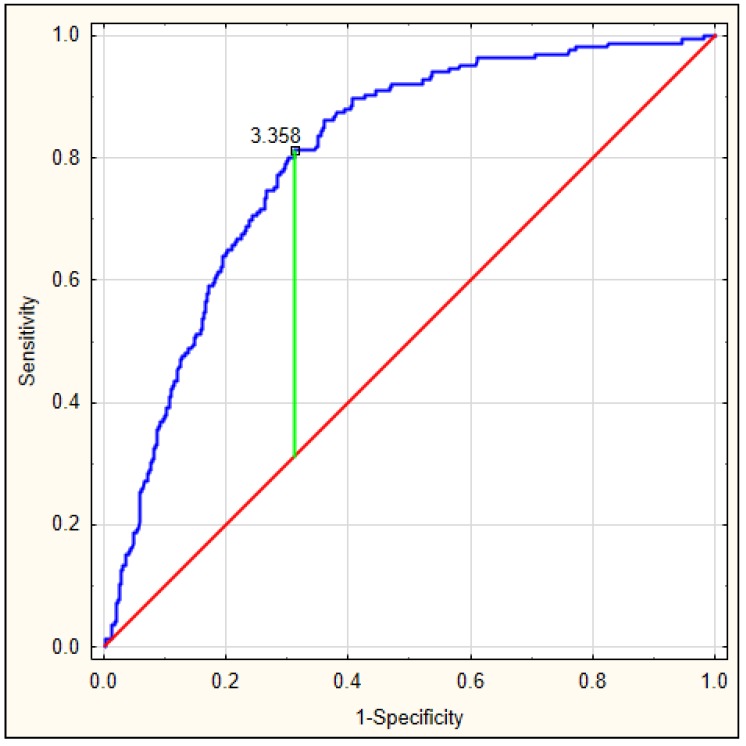
ROC analysis for DELIAS score based on the formula generated from the occurrence of early-onset delirium after AIS.

**Figure 4:**
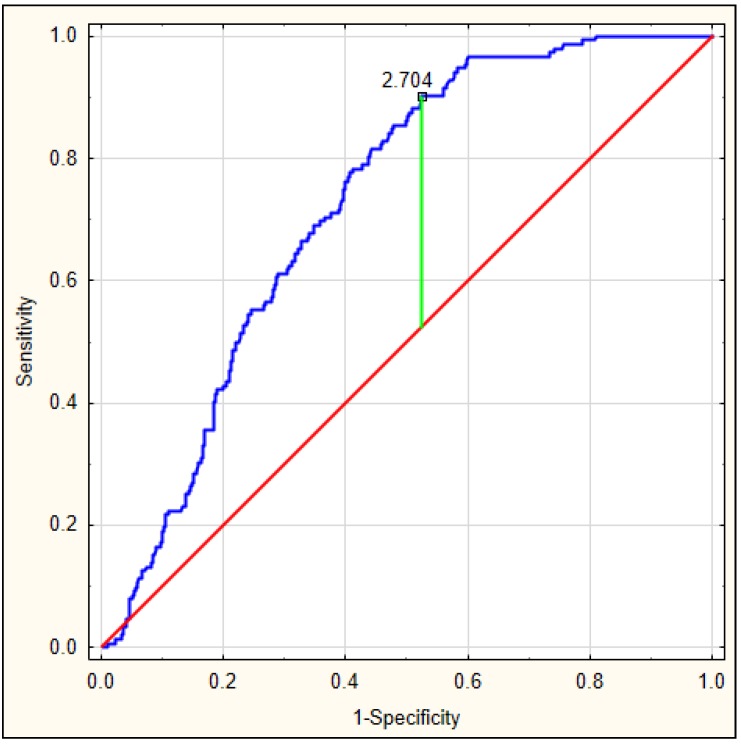
ROC analysis for DELIAS score for the occurrence of delirium until the fifth day from AIS.

On the basis of the obtained values, an ROC curve was determined. Figure 3 depicts the ROC curve for the DELIAS score, calculated based on a formula derived from data of patients with early-onset delirium. The optimal AUC was 0.801, *p* < 0.001, with very good sensitivity of 0.813 and specificity of 0.673. In this group, the DELIAS score can be regarded as a parameter with good predictive value for delirium prediction (Table 7). To verify the predictability value of the DELIAS score for the diagnosis of delirium, we performed an ROC analysis for delirium diagnosed up to the fifth day in the same group of patients. In this case, the AUC was 0.725, *p* < 0.001, with sensitivity of 0.901 and specificity of 0.477 (Figure 4). The DELIAS score had a good predictive value for early-onset delirium and moderate predictive value for delirium up to the fifth day from AIS.

Logistic regression analysis was performed for patients with early-onset delirium and for patients with delirium up to five days (Table 8). The analysis was made on the basis of the cut-off points of the obtained formula. For patients with early-onset delirium, a significant correlation was shown with the delirium score (OR = 8.976, *p* < 0.001). Similarly, a statistically significant correlation with the delirium score was demonstrated in patients presenting with delirium up to five days (OR = 7.744, *p* < 0.001).

**Figure 5:**
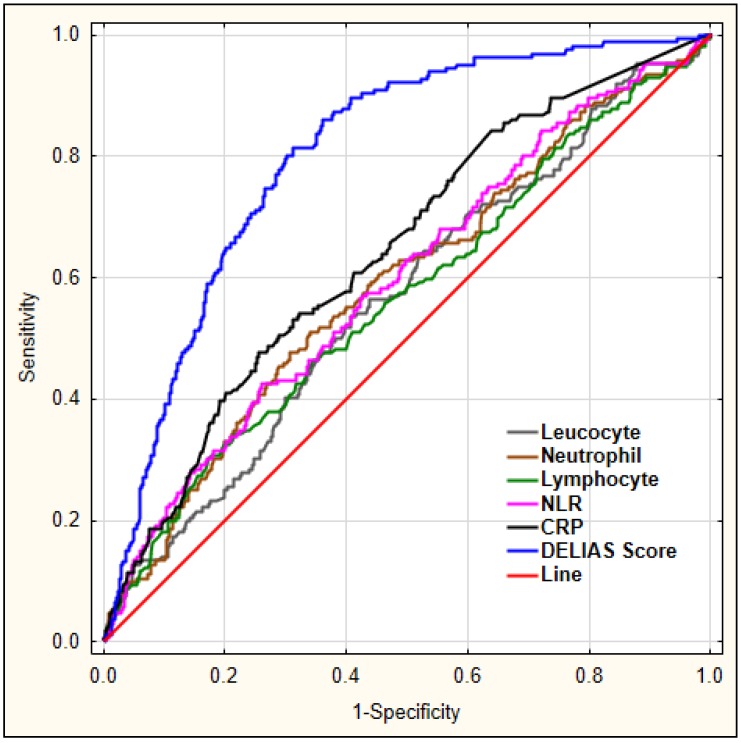
ROC analysis for chosen laboratory parameters for early-onset delirium. Legend: NLR—neutrophil-to-lymphocyte ratio, CRP—C-reactive protein.

Figure 5 shows a combination of factors and their predictive values for early-onset delirium as diagnosed using logistic regression, with DELIAS score showing the largest AUC.

## 4. Discussion

This study sought to determine if NLR is an early marker of delirium in patients with acute ischemic stroke. It is the largest prospective database evaluating the role of NLR as a prognostic marker for developing delirium in critically ill stroke patients. In the present study, we found that the mean levels of leukocytes and neutrophils were elevated, while those of lymphocytes were decreased in stroke patients with delirium. The NLR was elevated (6.39 ± 8.60 vs. 4.61 ± 5.61, *p* < 0.001), yet AUC for NLR was 0.597, indicating modest predictive ability. Even at the optimal cut-off of 4.86, the sensitivity was 42% and specificity was 74%, making it not an ideal standalone test for the detection of delirium. Despite the fact that the NLR AUC is not very discriminative given the relatively low AUCs, our investigation adds an important piece of evidence on the relationship between delirium and a dysfunction of immune effector cells. The data regarding neutrophil, lymphocyte, and the neutrophil-to-lymphocyte ratio in delirium had previously been limited to a couple of studies. 

The authors aimed to find a diagnostic score using easily accessible laboratory parameters to predict delirium after acute ischemic stroke. Adjusted multivariable logistic regression showed that, for NLR at the cut-off value >4.86, the OR was 1.875 (95% CI 1.314–2.675, *p* = 0.001). For C-reactive protein, this association was even stronger, and for CRP at the cut-off value >9.10, the OR was even higher than for NLR at 2.132 (95% CI 1.482–3.066, *p* < 0.001).

Therefore, the authors would like to underline that using clinical and laboratory factors combined in one score composed of age, NIHSS score, neurological findings, leucocyte count, NLR, and CRP is better at predicting early-onset delirium after acute ischemic stroke than any of the factors alone.

The findings of this study regarding NLR, white blood cells differential count, and CRP suggest that an inadequate response of the immune system and oxidative stress may play a role in the pathogenesis of delirium. Studies have shown that inflammatory markers and cytokines (i.e., neopterine, interleukin-8) can be detected in the cerebrospinal fluid of delirious patients [29,30,31]. Growing evidence suggests that neutrophils and lymphocytes are major effectors of acute inflammation [32]. The initial response in generalized stress, including stroke, is a nonspecific activation of the immune system, where the first-line response is the increase in neutrophil count and a decrease in the lymphocyte count [32,33]. This leads to a disruption of the neutrophil-to-lymphocyte ratio in peripheral circulation. 

In agreement with our findings, Egberts et al., in a pilot study on a small sample of patients, also found that elevated levels of NLR were associated with delirium in elderly patients [19]. In their study, 13 out of 86 patients were diagnosed with delirium and, using adjusted models, the authors showed that higher mean NLR values were found in patients with delirium (9.10 vs. 5.18, *p* = 0.003). The same study determined that the difference between the total number of leukocytes, neutrophils, and lymphocytes were not significant between patients with and without delirium. The authors suggested that only the right ratio, or a certain level of balance between neutrophils to lymphocytes, is important in the mechanism of delirium formation [19]. In their study group, the NLR had a weak correlation with the level of CRP. Our analysis showed a similar correlation between NLR and CRP in patients with AIS and delirium on admission to hospital. It has been shown that CRP has at least as strong an association with delirium after ischemic stroke as NLR, but it is not always available to the clinician. It would be more interesting if a combination of markers would help predict the presence of delirium.

As previously mentioned, stress-induced physiological immune system response is defined by an increase in the neutrophil count and a concomittant decrease in the lymphocyte count [34,35]. In delirium, elevated neutrophil count and NLR may reflect the degree of neuroinflammation and may be the basis of prediction of delirium by systemic inflammatory indicators [36]. NLR is a novel inflammatory biomarker proposed as a prognostic factor in different inflammatory diseases, including systemic lupus erythematosus (SLE), ulcerative colitis, and inflammatory arthritis, as well as in metabolic disorder diabetes mellitus or coronary artery disease [26,37]. NLR has previously been reported to be useful as a prognostic factor in acute ischemic stroke and transient ischemic attack [36,37]. Recently, Wu et al. reported that leukocytosis may be regarded as an early pathology of subarachnoid hemorrhage and that NLR may be used as a practical predictor for the occurrence of delayed cerebral ischemia in those patients [38]. A disturbance in peripheral blood lymphocyte subsets has been described to be associated with complications in high-risk vascular patients [39].

Lymphocytes are regarded as the mediators of chronic inflammation. Therefore, in the acute phase of inflammation, their numbers decrease initially, while the number of neutrophils increases; at a later stage, the lymphocyte level increases. This phenomenon may be a part of age-associated chronic inflammation of low-grade chronic immune system response to aging (inflammaging) and age-related diseases, such as delirium [40]. 

Lymphopenia and T-cell dysfunction are known predictors of mortality in ICU patients [36]. Lymphopenia may be regarded as a nonspecific yet readily available bedside marker of immunosuppression. Inoue et al. examined whether lymphopenia was associated with the development of acute brain dysfunction (delirium and/or coma) or 30-day mortality in general medical and surgical ICU patients. Their research regarding the number of lymphocytes in delirium showed that the reduced number of lymphocytes did not affect the number of days with delirium or in coma in patients in the intensive care unit [41]. The authors reported that patients with lower lymphocyte levels showed a trend toward higher chance of coma and delirium (*p* = 0.07), yet there was no significant relationship between lymphopenia and 30-day mortality [41].

The definite strength of our study is the large number of patients with delirium and AIS and its prospective data collection. The sample size of 1001 patients is larger than most studies reported in the literature to date on the risk of developing delirium in patients with AIS. The out-of-research setting of a district general hospital shows that easily obtainable markers (NLR, CRP) can be used in a general population as bedside indicators of acute brain dysfunction. We also used C-reactive protein as a control for inflammation. Results from the present study have derived a more advanced prediction formula (the DELIAS score) than other measures that have been reported in the literature on delirium in AIS. Our model could be presented in terms of a proposal for the construction of a score, that is, based on our mathematical approach, conclusions can be drawn to design, at a later date, a score that is easy to apply in any context. NLR as the biomarker may have an advantage over other measures reported in the literature, with a much simpler approach for wider practice than those reported in the past. 

Our study is not without limitations in terms of missing potential confounders, generalizability, or causative association between NLR and delirium. First, this is a single-center observation series in patients with acute ischemic stroke, and the results may not be applicable for other patients with delirium (ICU delirium, postoperative delirium, oncology patients). Second, we did not exclude patients using steroid therapy, and this may have had an influence on immune parameters. Third, we did not include white blood cell counts after admission as we were investigating the role of early factors available to guide the delirium diagnosis. This cut-off value for NLR was arrived at and not based on a standard value as no official standard cut-off for NLR currently exists. It would be necessary to perform an analysis regarding subtypes of white blood cells to determine the influence of immunity on different types of delirium in different settings. NLR seems to be a nonspecific marker and can be elevated in the context of a wide variety of conditions, such as cancer, diabetes, CAD, etc., even in the absence of delirium.

Despite these limitations, our study is the first study to evaluate the association between NLR and acute brain injury in patients with acute ischemic stroke. Further prospective multicenter studies in different settings are needed to evaluate the role of our preliminary conclusion.

## 5. Conclusions

In this study, we evaluated the association between laboratory markers, including NLR, and the presence of delirium in patients with acute ischemic stroke and found significantly higher values of NLR levels in patients with delirium compared to those without delirium. From this study, we can conclude that NLR can be used as a potential marker for prediction of early-onset delirium after acute ischemic stroke. On the basis of the combined laboratory and clinical parameters, a combination of parameters named the DELIAS score was determined, which showed the highest predictive values for delirium in the analyzed group of patients after ischemic stroke. The results of this study confirm that an inadequate response of the immune system may play a role in the pathogenesis of delirium after stroke.

## Figures and Tables

**Table 1:** Baseline characteristics of patients with acute ischemic stroke (AIS) with and without delirium.

Variables	Total (*n* = 1001)	No Delirium (*n* = 829)	Delirium (*n* = 172)	*p*-Value
**Demographic data**				
Age (years), Me (IQR)	71.00 (64.00–82.00)	70.00 (63.00–81.00)	79.00 (67.50–87.00)	<0.001
Gender (male), *n* (%)	523 (52.25)	445 (53.68)	78 (45.35)	0.047
BMI (kg/m^2^), Me (IQR)	26.03 (24.02–29.05)	25.95 (23.95–29.05)	26.64 (24.22–28.67)	0.496
Smoking, *n* (%)	417 (41.66)	343 (41.38)	74 (43.01)	0.689
**Comorbidities**				
Arterial hypertension, *n* (%)	869 (86.81)	721 (86.97)	148 (86.05)	0.744
Ischemic heart diseases, *n* (%)	258 (25.77)	198 (23.88)	60 (34.88)	0.003
Myocardial infarction, *n* (%)	107 (10.69)	88 (10.62)	19 (11.05)	0.868
Previous ischemic stroke, *n* (%)	222 (22.18)	172 (20.75)	50 (29.07)	0.017
Previous hemorrhagic stroke, *n* (%)	24 (2.39)	23 (2.77)	1 (0.58)	0.151
TIA last 30 days, *n* (%)	129 (12.89)	101 (12.18)	28 (16.28)	0.145
TIA earlier than last 30 days, *n* (%)	107 (10.69)	83 (10.01)	24 (13.95)	0.128
CT post-stroke lesions, *n* (%)	324 (32.37)	252 (30.4)	72 (41.86)	0.003
Acute renal failure, *n* (%)	9 (0.89)	5 (0.6)	4 (2.33)	0.083
Chronic renal failure, *n* (%)	143 (14.29)	109 (13.15)	34 (19.77)	0.024
Dialysis, *n* (%)	2 (0.19)	2 (0.24)	0 (0)	0.769
Impaired insulin tolerance, *n* (%)	48 (4.79)	29 (3.5)	19 (11.05)	<0.001
Diabetes (oral medications), *n* (%)	205 (20.48)	166 (20.02)	39 (22.67)	0.433
Diabetes (insulin), *n* (%)	129 (12.89)	101 (12.2)	28 (16.28)	0.146
Atrial fibrillation, *n* (%)	293 (29.27)	210 (25.36)	83 (48.26)	<0.001
AF paroxysmal, *n* (%)	61 (6.09)	51 (6.15)	10 (5.81)	0.866
AF persistent, *n* (%)	163 (16.28)	107 (12.91)	56 (32.56)	<0.001
ICA stenosis, *n* (%)	87 (8.69)	70 (38.67)	17 (47.22)	0.339
LICA, Me (IQR)	50 (0.0–50.0)	50 (0.0–50.0)	50 (0.0–50.0)	0.288
RICA, Me (IQR)	50 (0.0–50.0)	50 (0.0–50.0)	50 (0.0–50.0)	0.453
COPD	95 (9.49)	77 (9.29)	18 (10.47)	0.632
Peripheral vascular disease, *n* (%)	454 (45.35)	348 (41.98)	106 (61.63)	<0.001

Legend: *n*—number of patients, BMI—body mass index, TIA—transient ischemic attack, CT—computed tomography, AF—atrial fibrillation, ICA—internal carotid artery, LICA—left internal carotid artery, RICA—right internal carotid artery, COPD—chronic obstructive pulmonary disease, Me—median, IQR—interquartile range.

**Table 2:** Baseline neurology data for AIS patients with and without delirium.

Variables		No Delirium (*n* = 829)	Delirium (*n* = 172)	*p*-Value
NIHSS at admission, Me (IQR)		8.00 (4.00–14.00)	18.00 (12.00–21.50)	<0.001
Rankin score at admission, Me (IQR)		3.00 (1.00–4.00) 2.86 ± 1.56	5.00 (4.00–5.00) 4.37 ± 1.12	<0.001
Hemianopia, *n* (%)		242 (29.19)	127 (73.84)	<0.001
Dysphasia, *n* (%)		434 (52.35)	113 (65.70)	0.001
Brainstem stroke, *n* (%)		39 (4.70)	8 (4.65)	0.867
CT imaging-lesion, *n* (%)	No lesion	55 (6.63)	8 (4.65)	<0.001
	Lesion < 2.5 cm	378 (45.60)	29 (16.86)	
	Lesion > 2.5 cm	371 (44.75)	135 (78.49)	
	Lacunar stroke	25 (3.02)	0 (0)	
Cerebral artery involvement, *n* (%)	Anterior CA	186 (22.68)	9 (5.29)	<0.001
	Middle CA	513 (62.56)	140 (82.35)	
	Posterior CA	121 (14.76)	21 (12.35)	
Carotid artery stenosis, *n* (%)	No changes	29 (3.6)	3 (1.83)	<0.001
	Up to 50%	506 (62.86)	75 (45.73)	
	50–70%	169 (20.99)	63 (38.41)	
	>70%	50 (6.21)	6 (3.66)	
	Occlusion	51 (6.34)	17 (10.37)	
AIS treatment, *n* (%)	Conservative	638 (80.35)	145 (84.8)	0.178
	Thrombolysis	156 (19.65)	26 (15.20)	

Legend: NIHSS—National Institute of Health Stroke Scale, *n*—number of patients, CT—computed tomography, CA—cerebral artery, Me—median, IQR—interquartile range.

**Table 3:** Outcome for AIS patients with and without delirium.

Variables		No Delirium (*n* = 829)	Delirium (*n* = 172)	*p*-Value
Hospital LOS (days), Me (IQR) OR		9.00 (8.00–11.0) 0.977	10.00 (8.00–14.00) 1.023	<0.001
Time from stroke to death (days), Me (IQR) OR		29.00 (11.00–87.00) 1.002	25.00 (8.00–50.00) 0.998	0.161
Rankin score at discharge, Me (IQR) OR		2.00 (1.00–4.00) 0.664	5.00 (3.00–5.00) 1.506	<0.001
NIHSS at discharge, Me (IQR) OR		4.00 (2.00–12.00) 0.951	18.00 (6.00–29.00) 1.052	<0.001
Mortality at day 7, *n* (%) OR		41 (4.94) 0.279	27 (15.69) 3.579	<0.001
Mortality at day 30, *n* (%) OR		101 (12.18) 0.223	66 (38.37) 4.488	<0.001
Mortality at 1 year, *n* (%) OR		197 (23.76) 0.189	107 (62.21) 5.281	<0.001
Discharge	Death, *n* (%) OR	67 (8.08) 0.823	37 (21.51) 1.215	<0.001
	Home, *n* (%) OR	513 (61.88) 3.990	71 (41.28) 0.251	
	Nursing home, *n* (%) OR	63 (7.60) 1.200	29 (16.86) 0.834	
	Rehabilitation center, *n* (%) OR	175 (21.11) 3.221	30 (17.44) 0.310	
	Other, *n* (%) OR	11 (1.33) 1.215	5 (2.91) 0.823	

Legend: *n*—number of patients, OR—odds ratio, LOS—length of stay, NIHSS—National Institutes of Health Stroke Scale, Me—median.

**Table 4:** Laboratory values for AIS patients with and without delirium.

Variables	No Delirium (*n* = 829)	Delirium (*n* = 172)	*p*-Value
Leucocyte count (×10^9^/L); Me (IQR)	8.97 (7.33–11.27)	9.73 (7.67–11.91)	0.006
Neutrophil count (×10^9^/L); Me (IQR)	5.91 (4.47–7.92)	7.12 (4.99–9.52)	<0.001
Lymphocyte count (×10^9^/L); Me (IQR)	1.86 (1.38–2.46)	1.66 (1.13–2.31)	0.006
NLR; Me (IQR)	3.14 (2.06–5.09)	3.92 (2.44–7.87)	<0.001
CRP (mg/L); Me (IQR)	3.00 (1.10–9.69)	7.58 (2.25–30.00)	<0.001

Note: Laboratory markers are adjusted for age and sex. Legend: *n*—number of patients, NLR—neutrophil-to-lymphocyte ratio, CRP—C-reactive protein, Me—median.

**Table 5:** Details of the receiver operating characteristics (ROC) analysis for white cell values and C-reactive protein (CRP).

Variable	AUC	Youden’s Index	Cut-Off Value	Sensitivity	Specificity	*p*-Value
Leucocyte	0.566	0.13	9.53	0.535	0.592	0.006
Neutrophil	0.590	0.17	7.07	0.512	0.659	<0.001
Lymphocyte	0.567	0.13	1.23	0.308	0.823	0.008
NLR	0.597	0.16	4.86	0.424	0.739	<0.001
CRP	0.644	0.22	9.10	0.476	0.746	<0.001

Legend: AUC—area under the curve, NLR—neutrophil-to-lymphocyte ratio, CRP—C-reactive protein.

**Table 6:** Logistic regression analysis regarding inflammatory parameters according to the occurrence of delirium on admission in patients with acute ischemic stroke.

Independent Variable	Unadjusted		Adjusted	
	OR (95% CI)	*p*-Value	OR (95% CI)	*p*-Value
Leucocyte count (×10^9^/L)	1.064 (1.026–1.103)	<0.001	1.068 (1.028–1.110)	0.001
Neutrophil count (×10^9^/L)	1.047 (1.010–1.085)	0.012	1.043 (1.003–1.084)	0.035
Lymphocyte count (×10^9^/L)	0.918 (0.780–1.082)	0.307	0.959 (0.867–1.062)	0.424
NLR (mean)	1.036 (1.011–1.061)	0.005	1.025 (1.000–1.050)	0.049
NLR > 4.86	2.056 (1.463–2.890)	<0.001	1.875 (1.314–2.675)	0.001
CRP	1.007 (1.004–1.010)	<0.001	1.006 (1.003–1.010)	<0.001
CRP > 9.10	2.662 (1.888–3.753)	<0.001	2.132 (1.482–3.066)	<0.001

Note: Variables were adjusted according to age, sex, BMI, comorbidities, and baseline neurology. Legend: OR—odds ratio, CI—confidence interval, NLR—neutrophil-to-lymphocyte ratio, CRP—C-reactive protein.

**Table 7:** Details of the DELirium in Acute Ischemic Stroke (DELIAS) score for early-onset delirium (up to 24 hours) and delirium up to 5 days.

	AUC	Youden’s Index	Cut-Off Value	Sensitivity	Specificity	*p*-Value
DELIAS score for early-onset delirium (up to 24 hours)	0.801	0.50	3.358	0.813	0.673	<0.001
DELIAS score for delirium up to 5 days	0.725	0.38	2.704	0.901	0.477	<0.001

**Table 8:** Logistic regression analysis using DELIAS score to predict occurrence of delirium in patients with acute ischemic stroke.

	OR (95% CI)	*p*-Value
DELIAS score for early-onset delirium (up to 24 hours) for cut-off point > 3.358	8.976 (5.913–13.624)	<0.001
DELIAS score for delirium up to 5 days for cut-off point > 2.704	7.744 (4.531–13.234)	<0.001

## Data and Materials Availability

The dataset used and/or analyzed in this study are available upon a reasonable request from the corresponding author (A.S.), who was responsible for data management and statistical analysis.
